# Bionic design based on micro-nano structure of osteon and its low-velocity impact damage behavior

**DOI:** 10.1186/s40643-022-00600-9

**Published:** 2022-11-02

**Authors:** Yuxi Liu, Aihua Li, Yanhua Li, Song Chen

**Affiliations:** 1 School of Smart Health, Chongqing College of Electronic Engineering, Chongqing, 401331 China; 2 Department of Gastroenterology, Chongqing University Cancer Hospital, Chongqing, 400030 China; 3 Ministry of Education Key Laboratory of Child Development and Disorders, China International Science and Technology Cooperation Base of Child Development and Critical Disorders, Children’s Hospital of Chongqing Medical University, Chongqing, 400014 China; 4 College of Mechanical Engineering, Chongqing University of Technology, Chongqing, 400044 China

**Keywords:** Osteon, Mineralized fibril, Periodic helical structure, Impact resistance, Bionic composite

## Abstract

It is found that the osteon is composed of thin and thick lamellae which are periodic and approximately concentric, every 5 lamellae is a cycle, the periodic helix angle of mineralized collagen fibers in two adjacent sub-lamellae is 30°. Four bionic composite models with different fiber helix angles were established and fabricated according to the microstructure of mineralized collagen fibers in osteon. Based on the impact analysis of four kinds of bionic composite models, the effects of the fiber periodic spiral structure on the impact resistance and energy dissipation of multi-layer bionic composite were investigated. The analysis results show that the fiber helix angle affects the impact damage resistance and energy dissipation of multi-layer fiber reinforced composites. Among the 4 kinds of multi-layer composite models, the composite model with helix angle of 30° has better comprehensive ability to resist impact damage. The test results show that the impact damage area of the specimen with 30° helix angle is smallest among the 4 types of bionic specimens, which is consistent with the results of finite-element impact analysis. Furthermore, in the case of without impact damage, the smaller the fiber helix angle is, the more uniform the stress distribution is and more energy is dissipated in the impact process. The periodic spiral structure of mineralized collagen fibers in osteon are the result of natural selection of biological evolution. This structure can effectively improve the ability of cortical bone to resist external impact. The research results can provide useful guidance for the design and manufacture of high-performance and strong impact resistant bionic composites.

**Graphical Abstract:**

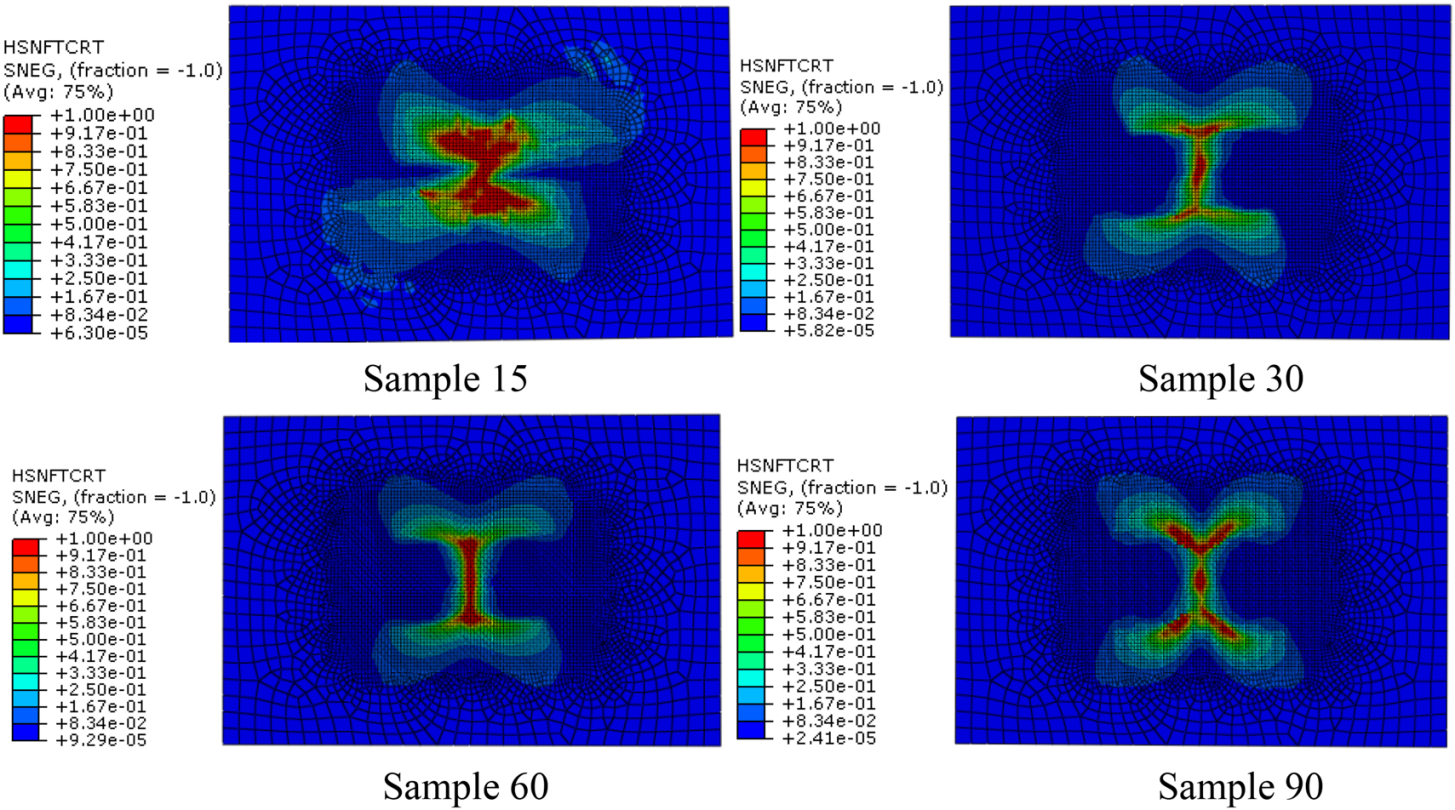

## Introduction

After hundreds of million-year of natural evolution, the unique biological structure of animals and plants have excellent mechanical properties which can achieve self-protection or resist natural enemies (Wang et al. 2020; Ingrole et al. 2021). With the rapid development of modern science and technology, composite materials with high strength, light weight (Rahimizadeh et al. 2021) and impact resistance (Wang et al. 2022, 2021) have a wide range of needs in the fields of aerospace, military, automobile and other fields (Sharma et al. 2022; Dong et al. 2022). Thus, the investigation of bionic composite materials can provide enlightenment for the design of exceptional impact resistant material and meet special requirement of engineering (Bhudolia and Joshi, 2018; Alizadeh and Ebrahimzadeh, 2022; Jiang et al. 2019a, b).

**Fig. 1:**
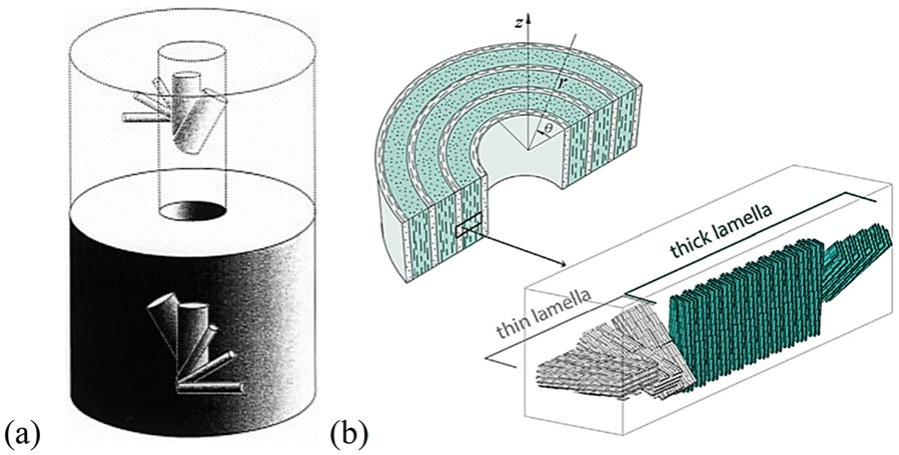
Microstructure schematic diagram of fiber arrangement in osteon. **a** Direction of fibers in adjacent sub-lamella and **b** structure and directions of thin and thick lamellae

As a typical biological material, bone plays an important role in supporting the animal body and protecting organs, which is composed of cortical bone and cancellous bone. From a structural viewpoint, cortical bone tissue can be considered as a composite material hierarchically structured at different scales. At the microscale, the mineralized collagen fibrils are grouped together in a certain direction to form lamellar structure, and the thickness is about of 3–7 μm. Furthermore, these lamellae are concentrical surround the haversian canal to compose osteons (Giner et al. 2014) and its diameter ranges from 50 to 500 μm (Currey 2012). At the nanoscale, cortical bone is mainly composed of organic phase (Dunlop and Fratzl, 2010) and inorganic phase, the organic phase is mainly formed by mineralized collagen fibrils (Hamed et al. 2010). Mineralized collagen fibers are ubiquitous in various biological materials and are mainly arranged helicoidally (Wang et al. 2018), and there are extensive researches on multilayer fiber bionic composite (Wei et al. 2020; Jiang et al.2019a, b). However, the mineralized collagen fibers in osteons have their own uniqueness, which are periodic helical arranged and every 5 sub-lamellae constitute a lamella. The arrangement of mineralized collagen fibers in adjacent sub-lamellae is shown in Fig. 1a (Giraudguille 1988; Liu et al. 2000), the 5 directions represent the fiber directions in five consecutive sub-lamellae in each lamella and the helical angle of 2 adjacent fibers is 30°.Giner et al. (Giner et al. 2014) drew a schematic diagram of the staggered structure of sub-lamellae of osteons and the fiber directions of the 5 sub-lamellae (Fig. 1b), and the 5 sub-layers are simplified to a thin layer and a thick layer (Vercher et al. 2014). The thickness of lamellae effects the stress distribution of osteon (Ismail et al. 2019), the microstructure of osteon affects the propagation path of microcracks in cortical bone (Gustafsson et al. 2019) and increase fracture toughness of cortical bone (Liu et al. 2019; Luo et al.^2022^). Furthermore, the osteon wall thickness decreases significantly in the aged group and X-FEM results indicate that the presence of osteon effects the critical stress intensity factor of bone (Yadav et al. 2021).

At present, the research on osteons mainly focuses on the microstructure of osteons (Yin et al. 2021; Vercher-Martínez et al. 2014; Reznikov et al. 2014), distribution of collagen fibers and osteocyte lacunae (Liu et al.^2017^, ^2019^). However, there is no report on the bionic composite material with periodic helical arrangement of fibers based on osteon. In this paper, the bionic composite models with periodic spiral structure were investigated based on the distribution of fiber in osteon. Four bionic composite models were constructed and fabricated for comparing and analyzing the effect of the periodic helical arrangement structure of fibers on the impact characteristics of composite*.* Then, low-velocity impact damage analysis and drop weight test were performed, the finite-element (FE) analysis is based on Hashin failure criterion and progressive damage analysis method, the influence of fiber laying method on the impact damage and energy dissipation ability of bionic composites were investigated.

## Materials and methods

### Bionic design and impact analysis

According to the helical structure of mineralized collagen fiber in osteon, a bionic composite model of periodic spiral structure model with the fiber helix angle increasing by 30º was constructed (Table 1, Sample 30). In addition, to compare and analyze the effects of fiber helix angle on the impact resistance of bionic composites, a composite material model of osteon-like with 0º/90º orthogonal model and fiber helix angle of 15º and 60ºmodels were constructed (Table 1). Then, the effects of different fiber arrangement structure on the impact resistance and energy dissipation capacity of the bionic composite were investigated based on finite-element (FE) analysis method.

**Table 1:** Models of multilayer fiber bionic composites

Bionic models	Fiber laying mode
Orthogonal model(Sample90)	[0/90]_6 s_
Small helix angle model(Sample15)	[0/15/30···/165]
Medium helix angle model(Sample30)	[0/30/60···/150]_2 s_
Large helix angle model(Sample60)	[0/60/120]_4 s_

**Fig. 2:**
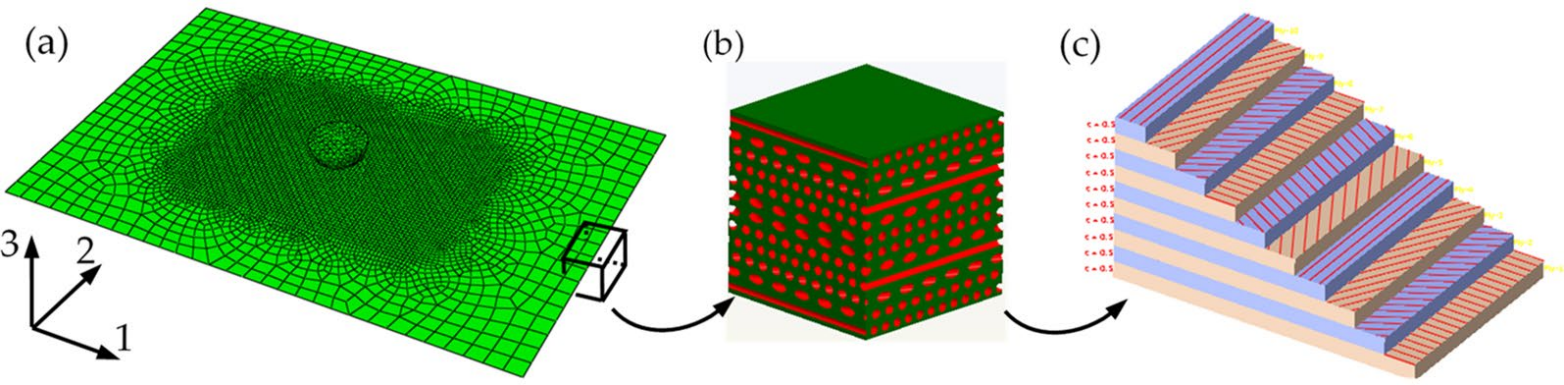
FE model of bionic composite. **a** Finite-element model for impact analysis of periodic helical bionic structure, **b** local enlarged view of the model (red represents fiber and green represents matrix), and **c** direction of fiber arrangement of composite

It is assumed that the thickness of each sublayer in the helical structure is same and the layers are well-integrated among themselves. Then, according to the periodic spiral structure of the fibers in osteon, a 12-layer periodic spiral structure bionic model was constructed in ABAQUS (Fig. 2). The geometric size of the model is 150 mm × 100 mm × 6 mm (standard thickness in ASTM-D-7136: 4 ~ 6 mm), and the single layer thickness is 0.5 mm. The thickness of bionic composite model is 6 mm in Table 1, and the thickness of each sublayer is equally distributed. The mechanical performance parameters of the composite material models used in FE analysis are shown in Table 2 (Karakuzu et al.^2010^; Hansen et al.^1999^; Ekhtiyari et al.^2022^).

**Table 2:** Characteristic parameters of composite material

Material characteristics	Value	Material characteristics	Value
Density*ρ*/(kg/m3)	1830	Transverse compressive strength*Y*_c_/MPa	124
Longitudinal modulus*E*_11_/GPa	40.51	In-plane shear modulus *G*_12_/GPa	3.1
Transverse modulus*E*_22_/GPa	13.96	In-plane shear strength, *S*_12_/GPa	69
Poisson's ratio*ν*_12_	0.22	Interlaminar shear strength, *S*_1_/MPa	38
Longitudinal tensile strength*X*_t_/MPa	783.3	Longitudinal critical energy release rate *G*_cr,L_/(kN·m^−1^)	40
Longitudinal compression strength*X*_c_/MPa	298	Transverse critical energy release rate *G*_cr,T_/(kN·m^−1^)	0.3
Transverse tensile strength*Y*_t_/MPa	64		

The impact analysis was performed on the four bionic material models using drop-weight impact testing. In the analysis process, the impact energy of 20 J was selected according to the dimensions of the bionic composite model and the drop weight impact test of fiber reinforced composite (Anuse et al. 2022), the mass of the drop-weight is 2 kg, and the critical contact velocity is 4.47 m/s. The elastic modulus *E* and Poisson's ratio *ν* of the punch are 210GPa and 0.3, respectively. The deformation of the punch is ignored in the process of impact analysis, that is, the punch is constrained as a rigid body. The shape of the punch tip is hemispherical with a diameter of *ϕ*16 mm. A reference point was chosen on the punch and added a mass point. Impact velocity was applied on the mass point, that is, the impact energy is applied to the punch according to the formula *E* = *mv*^2^/2. Then, the impact responses of the four material models were studied under the same impact energy.

The impact analysis model of periodic helical bionic structure with fiber helix angle of 30° is shown in Fig. 2a, the local enlarged view of the model and the distribution direction of the fibers in each layer are shown in Fig. 2b, c. For multi-layer composite structure analysis, the 3D shell element model can not only save the calculation cost, but also obtain higher accuracy. Therefore, the four models constructed in this analysis are modeled by 3D shell element, and the element type and size are the same. In addition, due to the severe mesh deformation in impact center area, to improve the calculation accuracy, the mesh in the impact center area was refined in the meshing stage. Then, the mesh size is transferred to the boundary region to ensure the mesh quality and reduce the calculation time.

There are 6 292 nodes and 6 275 hyperbolic shell elements (S4R) with large strain, reduced integral and sand leakage control. According to the specimen requirements of ASTM-D-7136 for drop-weight impact test, full restraint was applied to the four sides of the model. Due to the hard contact between the punch and the composite plate in impact process which will cause the failure of contact element, thus the ordinary hard contact algorithm is adopted. During the impact process of the model, the failure degradation of the element is based on the Hashin failure criterion, and the fiber is 0º along the direction 1 and 90º along the direction 2 (Fig. 2a).

### Material failure criterion of impact analysis

There are many kinds of damage in multi-layer composite under low-speed impact load, which are mainly divided into in-plane damage and interlayer damage. The in-plane damage of composites mainly includes fiber fracture and matrix crack, and the interlaminar damage mainly refers to delamination failure between sublayers. Energy-based damage evolution was adopted for the interlaminar interface of the multi-layer composite. Because the material is squeezed under impact load, the local interlaminar damage will not cause complete damage of the material. Therefore, this paper focuses on the comparison and analysis of the in-plane damage of materials with different fiber stacking methods.

#### Damage failure criterion

The Hashin criterion is used to simulate the impact damage of multilayer composite, which can predict the in-plane damage process of multilayer composite, including fiber tensile failure, fiber compression failure, matrix tensile failure and matrix compression failure. Due to the Hashin failure criterion can accurately determine various damage failure modes and is simple and effective, it has been widely used in practice. The combination of Hashin failure criterion and stiffness degradation criterion can simulate the progressive damage process of composite materials and can be easily realized.

The expression of Hashin failure criterion is as follows:

Fiber tensile failure (${\sigma }_{11}\ge 0$)

1 $${\left(\frac{{\sigma }_{11}}{{X}^{T}}\right)}^{2}+{\left(\frac{{\sigma }_{12}}{{S}^{L}}\right)}^{2}=1$$

Fiber compression failure (${\sigma }_{11}<0$)

2 $${\left(\frac{{\sigma }_{11}}{{X}^{C}}\right)}^{2}=1$$

Tensile failure of matrix (${\sigma }_{22}\ge 0$)

3 $${\left(\frac{{\sigma }_{22}}{{Y}^{T}}\right)}^{2}+{\left(\frac{{\sigma }_{12}}{{S}^{L}}\right)}^{2}=1$$

Matrix compression failure (${\sigma }_{22}<0$)

4 $${\left(\frac{{\sigma }_{22}}{2{S}^{T}}\right)}^{2}+\left[{\left(\frac{{Y}^{C}}{{2S}^{T}}\right)}^{2}-1\right]\cdot {\left(\frac{{\sigma }_{22}}{{Y}^{C}}\right)}^{2}+{\left(\frac{{\tau }_{12}}{{S}^{L}}\right)}^{2}=1$$

where *X*^*T*^ is the longitudinal tensile strength of the single layer; *X*^*C*^ is the longitudinal compressive strength of the single layer; *Y*^*T*^ is the transverse tensile strength of the single layer; *Y*^*C*^ is the transverse compressive strength; *S*^*L*^ is the longitudinal shear strength; *S*^*T*^ is the transverse shear strength; *σ*_11_, *σ*_22_ and *τ*_12_ are effective stress tensor components.

#### Material degradation criterion

Material degradation (i.e., material stiffness degradation) means that when the element satisfies certain failure criteria in the finite-element model, the elements in the model will be damaged (including matrix failure, fiber failure and delamination). According to these different damage modes, the material properties of the damage element in the model need to be given new values according to certain rules, then a new material model obtained.

In the finite-element progressive damage analysis, there are many methods to degrade the stiffness of the element. In this research, the material parameter degradation mode (Zhang et al. 2021) was used to degrade the stiffness of damaged area, and different in-plane damage modes correspond to different degradation schemes, as shown in Table 3.

**Table 3:** Material stiffness degradation criterion

Damage mode	Degradation criteria
Matrix tensile failure(*σ*_22_ ≥ 0)	*Q*_0_ = 0.2*Q*(*Q* = *E*_22_, *G*_23_, *ν*_12_)
Matrix compression failure(*σ*_22_ < 0)	*Q*_0_ = 0.4*Q*(*Q* = *E*_22_, *G*_23_, *ν*_12_)
Fiber tensile failure(*σ*_11_ ≥ 0)	*Q*_0_ = 0.07*Q*(*Q* = *E*_11_, *G*_23_, *ν*_12_)
Fiber compression failure(*σ*_11_ < 0)	*Q*_0_ = 0.2*Q*(*Q* = *E*_11_, *G*_23_, *ν*_12_)

### Bionic composite fabrication and test

#### Specimen fabrication

**Fig. 3:**
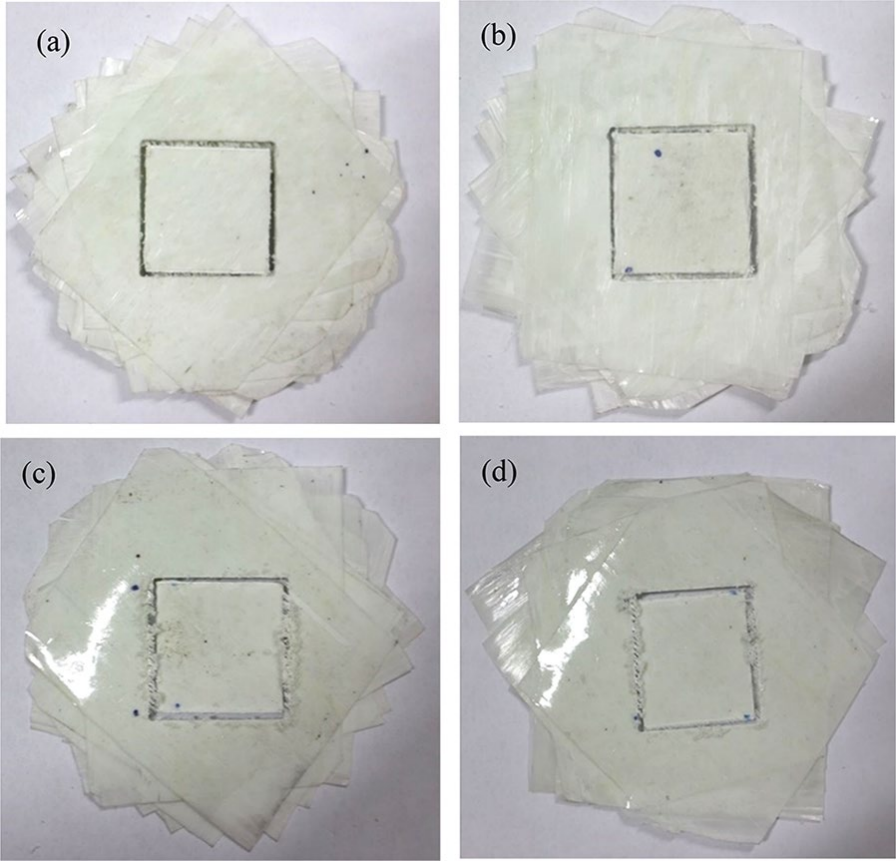
Biomimetic composite laminates with different helix angles. **a** Sample with fiber helix angle of 15º, **b** sample with fiber helix angle of 30°, **c** sample with fiber helix angle of 60°, and **d** sample with fiber helix angle of 90°

According to the designed four kinds of fiber periodic spiral structure models, unidirectional glass fiber prepreg (glass fiber/epoxy resin, model: G 12,500) was used to fabricate bionic spiral structure composite laminates. The fiber ratio of unidirectional glass fiber prepreg is 125 g/m^2^, the resin content is 33% (included 33wt% of resin), and the thickness of single layer is 0.1 mm. The fabrication processes of the composite laminate with fiber periodic helix ply structure are as follows:

1. The prepreg was cut into a square of 120 mm × 120 mm.
2. The cut prepreg was arranged periodically at the angles of 15°, 30°, 60° and 90° (Fig. 3). The number of layers is 12.
3. The prepreg lamination is put into the hot press mold, and the hot press (hot press model: hy61zf) is used for hot press curing.

The hot pressing process as follows:

1. The initial pressure is 2t, and the temperature is raised to 200℃;
2. After holding the pressure for 5 min, the pressure dropped to 1t;
3. The composite laminate with bionic spiral structure can be obtained by keeping the pressure for 120 min and cooling naturally.

#### Drop weight test

The composite laminates with bionic spiral ply structure were made into drop weight impact test specimens (size: 50 mm × 50 mm). Then, the tests were conducted according to the test method of ASTM- D-7136 for drop-weight impact test. The model of impact testing machine is XH-2000, the diameter of rigid body punch is 8.5 mm and the dimension of punch tip is 4 mm, the mass of the drop-weight is 2 kg, the dropheight is 1 m. Thetotal impact energy (J) of a specimen was computed bythe equation *E* = *MgH*, where *M* is theweight of the hammer (kg), *g* is 9.8 m/s^2^, and *H* is the height of drop weight (m).

## Results and discussion

### Effects of laying mode on damage resistance

When the multi-layer composites with different fiber arrangements are subjected to external impact, the in-plane damage is quite different. To compare and analyze the impact of different fiber stacking methods on the impact characteristics, the same boundary conditions were used for the four models in the impact analysis process, that is, the impact load and boundary constraints are the same.

#### Fiber compression failure

**Fig. 4:**
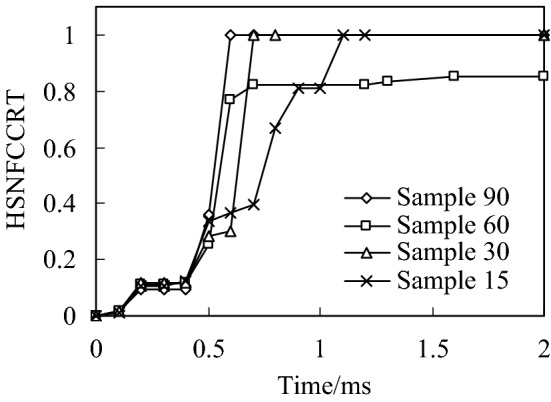
Relationship of fiber compression failure and impact time history

When the impact energy is 20 J, the initial failure of fiber compression and the failure distribution nephogram of the four kinds of bionic composite models in the impact process are shown in Figs. 4 and , respectively. According to the previous analysis of fiber compression failure criterion, the fiber compression failure occurs when the failure criterion is equal to 1, the fiber compression failure occurs when the failure criterion is greater than 1. It can be seen from the analysis results (Fig. 4) that with the increase of impact energy, the fiber compression failure occurs first in the orthogonal material model (sample 90), followed by the sample 30 model, and finally the sample 15 model. However, there is no obvious fiber compression failure in the sample 60 model under the impact load. In addition, it can be seen from Fig. 4 that although the fiber compression failure exists in the sample 15, sample 30 and sample 90 models, there is a significant difference in the failure initiation time.

**Fig. 5:**
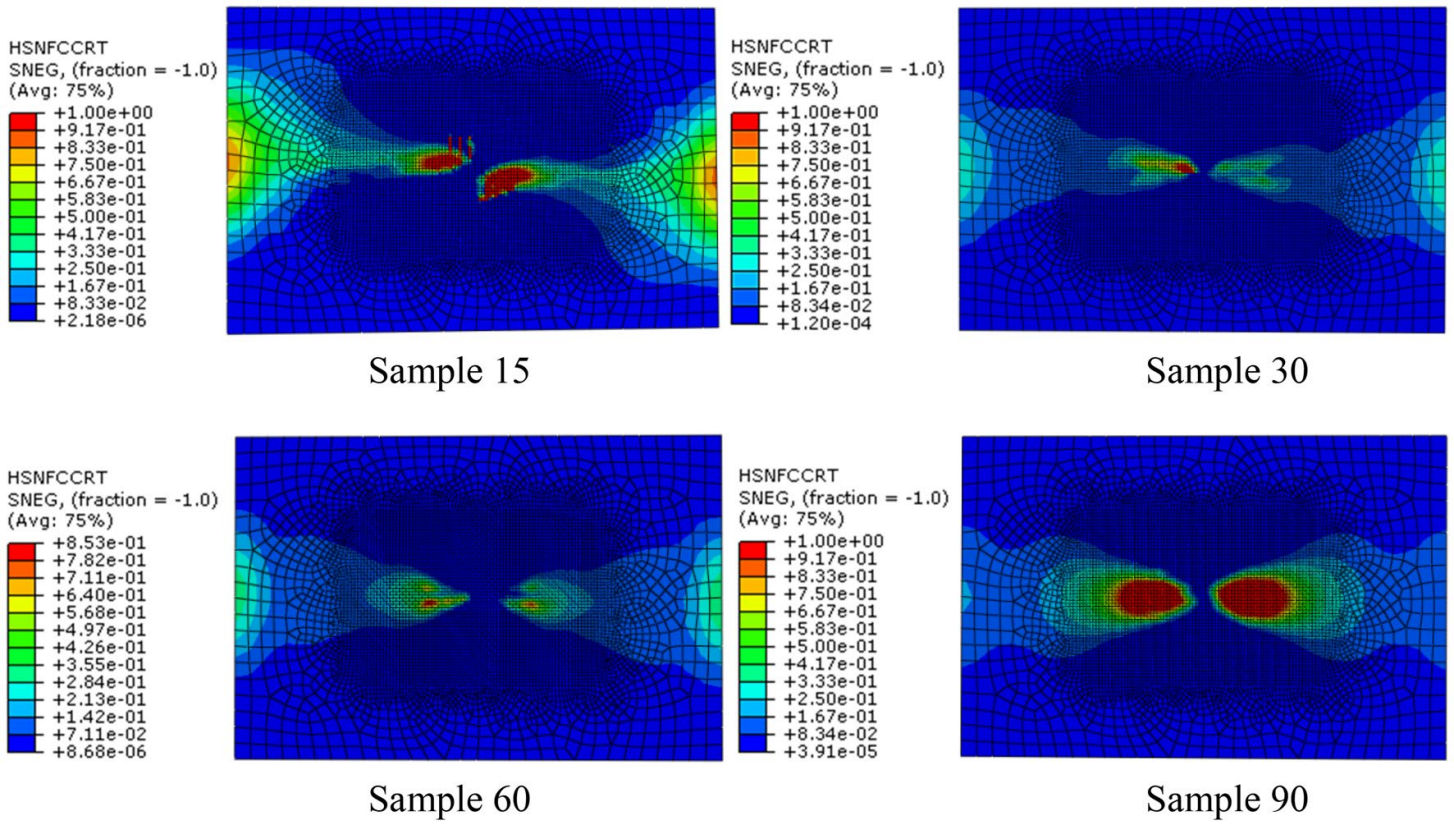
Distribution nephogram of fiber compression failure of four bionic models with different helix angles when impact energy is 20 J

In the distribution nephogram of fiber compression failure, the red area represents the part of fiber compression failure. It can be seen from the failure distribution nephogram (Fig. 5) that the fiber compression failure ratio in the sample90 model is the largest, followed by the sample15 model, and the failure proportion in the sample30 model is the smallest, which indicates that the collagen fiber arrangement structure with a helix angle of 30° is helpful to enhance the compression resistance ability of osteon.

The above analysis results show that the fiber stacking mode directly affects the compression failure of the fiber. By adjusting the fiber stacking mode in the multi-layer composite, the initial time of fiber compression failure and the ratio of fiber compression failure can be effectively improved.

#### Fiber tensile failure

**Fig. 6:**
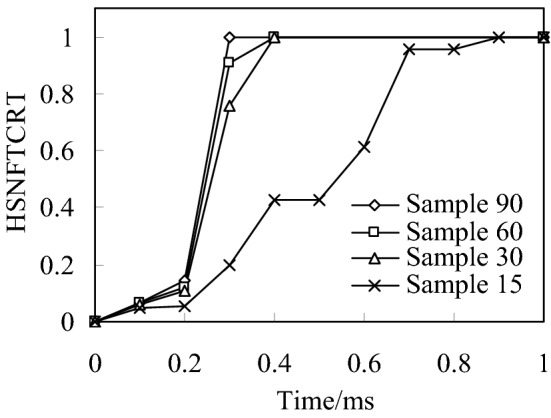
Relationship of fiber tensile failure and impact time history

Figure 6 shows the time history of fiber tensile initial failure of four bionic composite models during impact process. It can be seen from the analysis results that under the same impact load, the model of sample90 first appears fiber tensile failure. The second model is sample60 and sample30, but there are some differences in their time histories. Finally, the sample15 model, and the fiber tensile failure time of sample15 is much later than the first 3 models, which shows that the fiber has strong ability to resist tensile crack initiation in sample 15. Followed by the sample60 and sample30 models, but there are also certain differences in their time history. Finally, the sample15 model, and the fiber tensile failure time of sample 15 is much later than the first three models, which shows that the fiber has strong ability to resist tensile crack initiation in model sample15. From the tensile failure modes of four fiber reinforced models can be seen that the fibers failure in the bionic model are similar with the impact damage of multilayered fiber reinforced composites (Sayam et al. 2022; Veerakumar et al. 2021), this also shows the correctness of the finite-element simulation analysis.

**Fig. 7:**
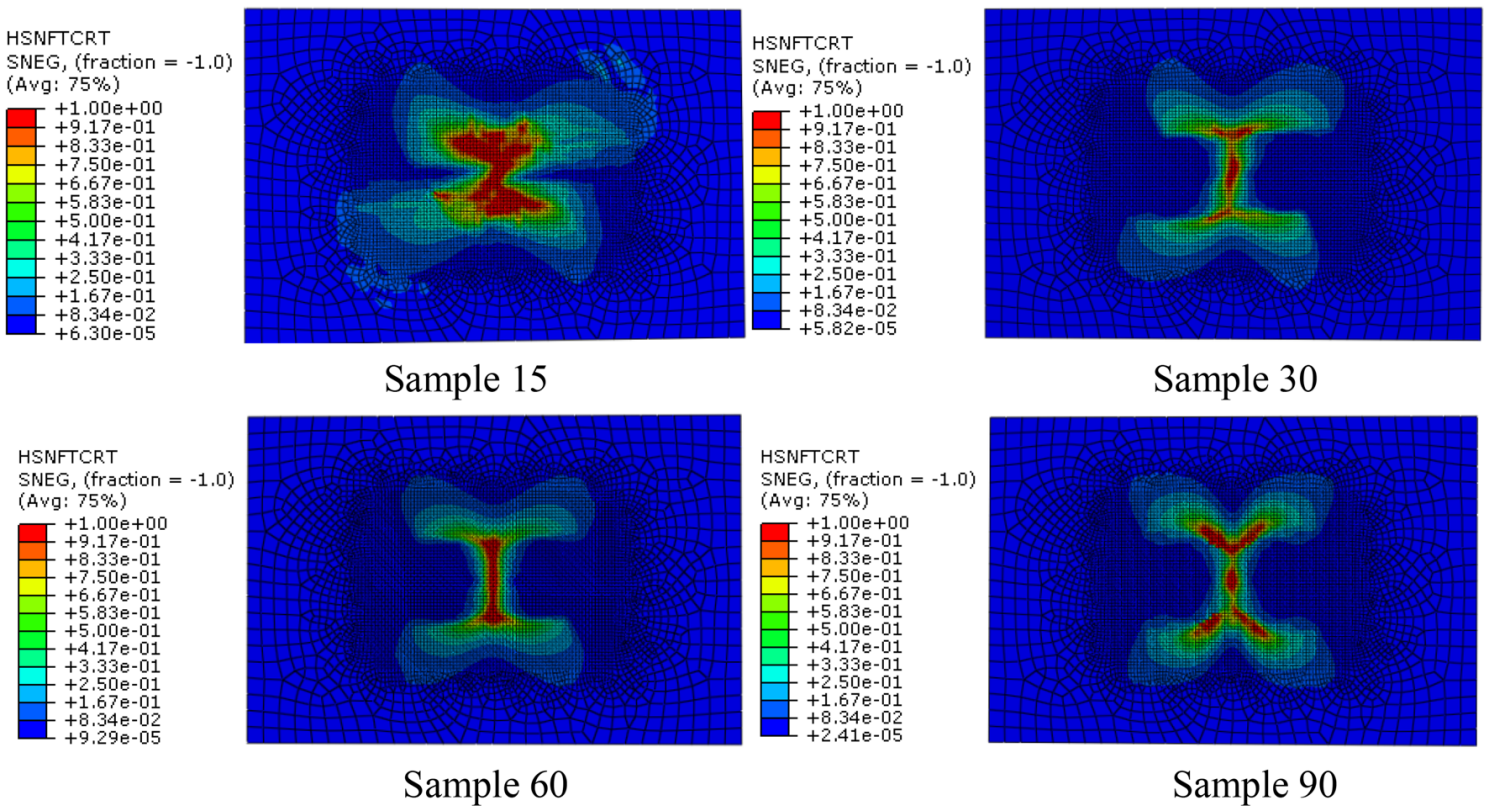
Distribution nephogram of fiber tensile failure of four bionic models with different helix angles when impact energy is 20 J

It can be seen from the distribution nephogram of fiber tensile failure (Fig. 7) that the proportion of fiber tensile failure increases in three models with fiber helix angle from 30° to 90°. However, the fiber tensile failure ratio is the largest when the helix angle is 15°, which indicates that the smaller the helix angle, the stress of the fiber in the model is more uniform. Once the failure occurs, the damage ratio will increase rapidly. The above analysis results show that the tensile strength of the multi-layer composite with fiber helix angle of 30° is stronger. Simultaneously, it also shows that the helix structure of collagen fibers with a helix angle of 30º in osteon helps to enhance the tensile strength of cortical bone.

#### Matrix compression failure

**Fig. 8:**
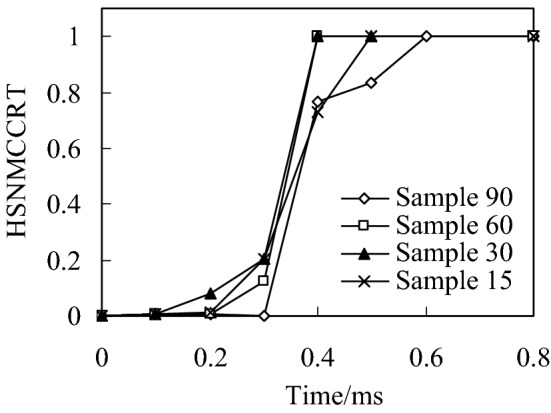
Relationships of matrix compression failure and impact time history

**Fig. 9:**
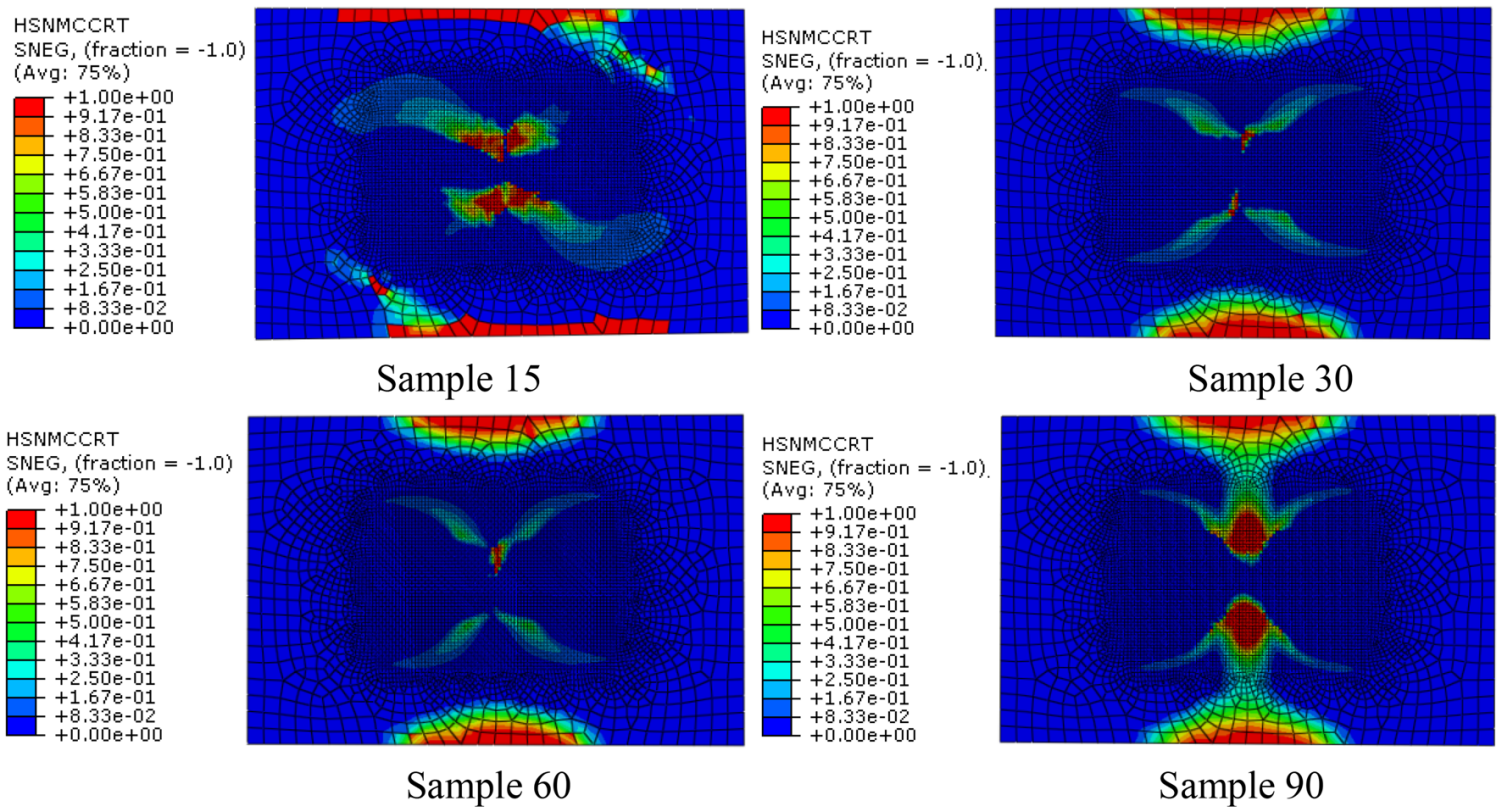
Distribution nephogram of matrix compression failure of four bionic models with different helix angles when impact energy is 20 J

Under the load of 20 J impact energy, the matrix compression failure history with time is shown in Fig. 8. It can be seen from Fig. 8 that matrix compression failure occurs first in sample30 and sample60, followed by sample15, and finally by sample90. Figure 9 shows the matrix compression failure distribution nephogram of the four models at the end of the impact. It is can be seen that the sample90 is the last matrix compression failure from the analysis results, the failure area is the largest at the end of the impact. In addition, by comparing the failure nephogram of the four models, can be seen that failure ratio of sample15 and sample90 is larger, while that of sample30 and sample60 are smaller. Furthermore, it can be seen from the failure areas of the four models that the failure areas are mainly concentrated in the center area and upper and lower edge areas of the model, and the upper and lower edges are the first to fail. According to the research results of Bunea et al. (Bunea et al. 2021) to impact response of fabric reinforced epoxy composites, edge failure is mainly due to the edge of the reinforced composite is pulled off. The results show that the stacking mode also affects the matrix compression failure for multi-layer fiber reinforced composites. Furthermore, when the multi-layer fiber reinforced composites are subjected to external impact, too large or too small fiber helix angle is not conducive to resistance compression failure of matrix.

#### Tensile failure of matrix

**Fig. 10:**
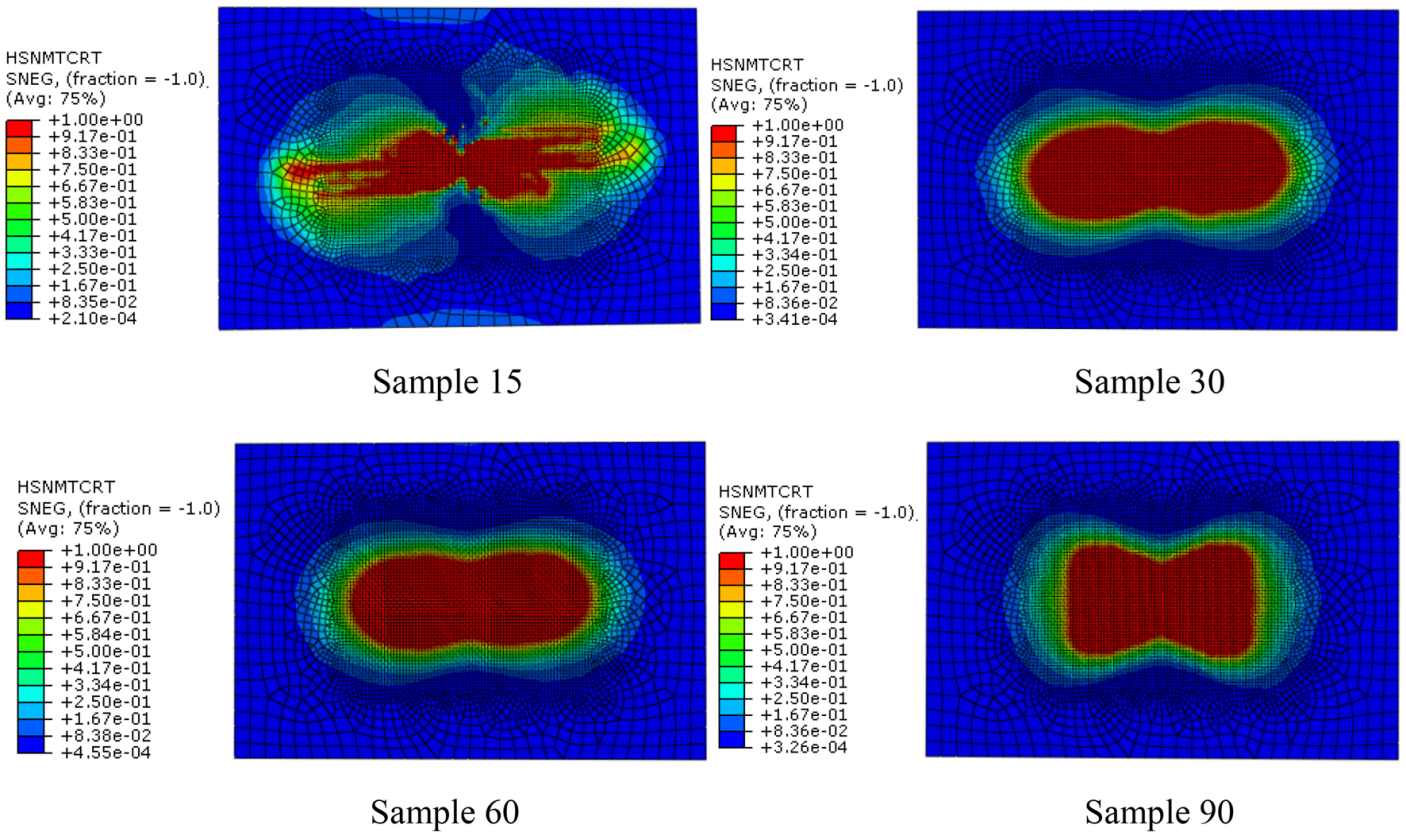
Distribution nephogram of matrix tensile failure of four bionic models with different helix angles when impact energy is 20 J

According to the impact damage analysis results of the four models, can be seen that matrix tensile failure is the first to occur in the four models. When the impact energy is 20 J, the matrix tensile failure distribution of the 4 models is shown in Fig. 10. Because the mechanical properties of the matrix in the multi-layer fiber reinforced composites are much lower than that of the reinforced phase, the matrix is usually damaged first when it is subjected to external force, so the analysis results in this section are consistent with the actual situation. In addition, according to the matrix tensile failure distribution nephogram of the four models, can be seen that matrix compression failure distribution nephograms of sample30, sample60 and sample90 are similar, but the tensile failure areas of the matrix are not different. Compared with the other three models, the tensile failure area of the matrix in sample15 is the smallest, and the failure distribution nephogram is obviously different from the other three models. It can be seen from the tensile failure distribution of the matrix that the failure area of the matrix is roughly in the shape of a "peanut", and the collective damage results obtained from composite impact test studies are also in this shape (Pingulkar et al. 2021; Zhang et al. 2020), which shows the correctness of the numerical model and analytical calculation established in this paper. The analysis results show that under the same material composition, boundary conditions and impact load, the fiber layering method also affects the tensile failure of the matrix, and the small fiber helix angle can make the matrix stress distribution more uniform, which is helpful to enhance the tensile strength of the matrix.

### Effects of laying mode on energy dissipation

Energy is an important factor in low-velocity impact test. The difference between the impact energy and the kinetic energy of punch pin at the end of the impact is defined as the energy dissipated by the composite plate during the impact. The "dissipated energy" defined here also includes other energy consumption during impact process, such as strain energy released by sandwich plate, kinetic energy of sandwich plate, strain energy released by punch, consumption of viscous damping and friction. The energy is difficult to accurately count and is very small, so it can be ignored. In addition, compared with multilayer composite materials, the punch is regarded as a rigid body, and its strain energy can also be ignored. In addition, compared with the multilayer composite, the punch is regarded as a rigid body, and its strain energy can be ignored.

The calculation method of impact energy used in this paper as follows: the kinetic energy of the punch at moment of contact is regarded as the impact energy. Therefore, the actual impact energy *E*_*impact*_ and the dissipated energy of composite plate *E*_*dissipated*_ are defined as follows:

5 $${E}_{impact}=\frac{1}{2}{mv}_{0}^{2}$$

6 $${E}_{dissipated}=\frac{1}{2}{mv}_{0}^{2}-\frac{1}{2}{mv}_{t}^{2}$$

where *m* is the mass of the punch, in this study *m* = 2 kg (the mass of different sizes of punch is slightly different in actual experiment); *g* is the acceleration of gravity; *h* is the distance of the punch relative to the upper surface of the test model before release; *v*_0_ and *v*_*t*_ are the velocities of the punch at moment of contact and separation between the punch and the upper surface of the model, respectively.

**Fig. 11:**
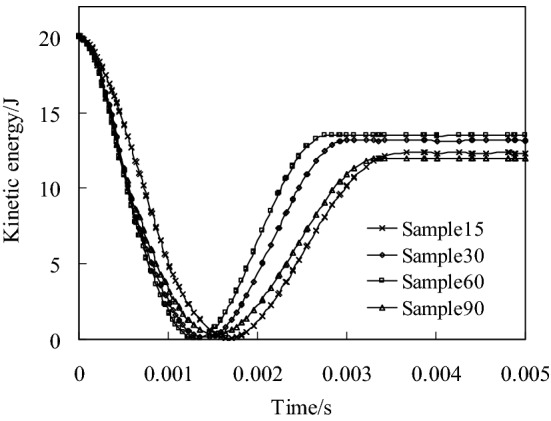
Change history of kinetic energy of the four models when the impact energy is 20 J

**Fig. 12:**
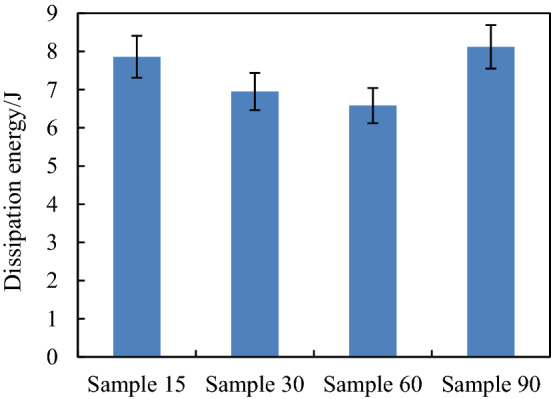
Dissipated energy of the four different models when the impact energy is 20 J

When the impact energy is 20 J, the kinetic energy change history of the four models is shown in Fig. 11. It can be seen from Fig. 11 that when the punch contacts with the composite plate, the velocity gradually decreases to zero, and then it is ejected and detached from the composite plate. The kinetic energy when the punch separates from the plate is the residual energy of impact. According to the kinetic energy change curve in Fig. 11, the energy dissipated by the four models in the impact process is shown in Fig. 12.

It can be seen from Fig. 12 that when the helix angle of the fiber decreases from 60° to 15°, the energy dissipated increases by 5.94% for sample30 and 18.37% for sample15 compared with sample60.The energy dissipated by the sample90 model is the largest. According to the above analysis of the four models can be known, this is due to more matrix and fiber damage in the sample90.

### Bionic specimens test results

**Fig. 13:**
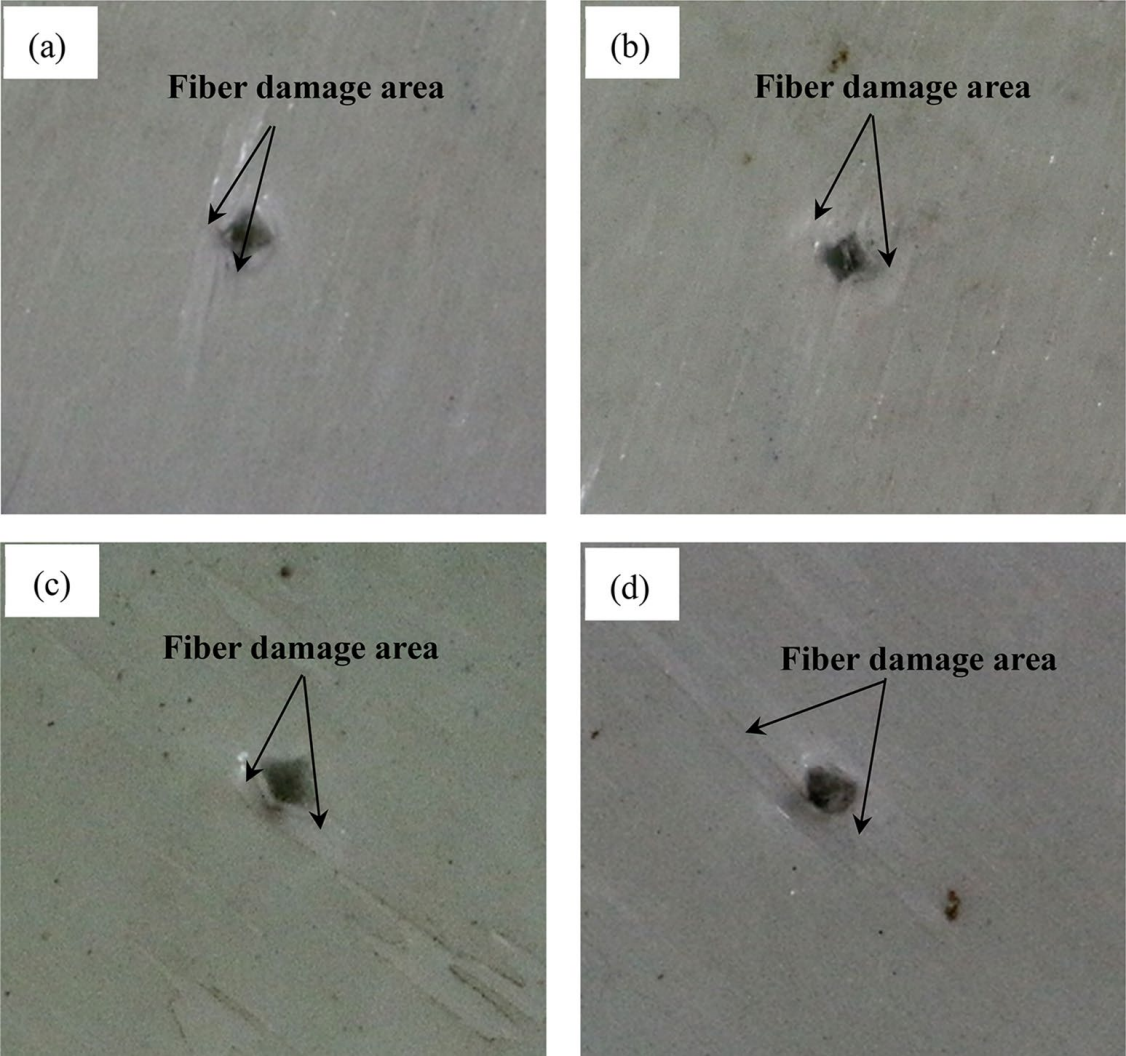
When the drop-weight is 2 kg and the drop-height is 1 m, the impact failure state of four bionic specimens. **a** 15°, **b** 30°, **c** 60°, **d** 90°

Figure 13 shows the failure mode of layered bionic specimens after impact. It can be seen from Fig. 13 that bionic composite laminates with different helix angles have varying degrees of damage under the same impact load. The results show that the impact damage area of the sample with 30° helix angle is smallest among the four types of bionic specimens. It can be concluded that the bionic composite laminate with fiber helix angle of 30° has a better ability to resist impact damage, which is consistent with the results of finite-element impact analysis, which also shows the correctness of the FE analysis. By comparing the impact damage test results of this bionic material with those of general composite materials, which can be seen that the impact damage mode of the bionic composite with fiber helix angle of 15° and 30° is similar with that of fabric reinforced epoxy composites of various angles (Bunea et al. 2021). However, the damage area of test specimens 60° and 90° are concentrated in the contact area between the punch and the specimen and the area along the 45° direction of the specimen, and its damage mode is similar with that of F_GnPs_ composite 0.1 wt.% (Pingulkar et al. 2021).

## Conclusions

Four kinds of bionic composite models with different fiber helix angles were established according to the microstructure characteristics of osteon, and the progressive damage analysis method was used to investigate the impact damage and energy dissipation capacity of the four models. Then, the bionic structure with different helix angles were fabricated and tested. The conclusions are described as follows:

1. The impact resistance of multi-layer fiber reinforced composites is affected by the way of fiber laying. The analysis results show that the model with a fiber helix angle of 30° has the best comprehensive ability to resist impact damage.
2. The smaller fiber helix angle can make the matrix stress distribution more uniform, which is helpful to enhance the tensile failure resistance of matrix. The tensile failure area of the matrix in sample15 is the smallest under the same boundary conditions and impact load.
3. The size of the fiber helix angle affects the energy dissipation capacity of the multilayer fiber reinforced composite. In the case of without impact damage, the smaller the fiber helix angle, the more energy dissipated in impact process.
4. The test results show that the impact damage area of the specimen with 30° helix angle is smallest among the four types of bionic specimens and has a better ability to resist impact damage, which is consistent with the results of finite-element impact analysis.
5. The helical structure of mineralized collagen fibers in osteon is the result of natural selection of biological evolution. This structure can effectively improve the ability of cortical bone to resist external impact. The research results can provide useful guidance for the development of high-performance bionic composite materials.

## Data Availability

All data generated and analyzed during this study are included in this published article.

## References

[CR1] Alizadeh SR, Ebrahimzadeh MA (2022). O-substituted quercetin derivatives: structural classification, drug design, development, and biological activities, a review. J Mol Struct.

[CR2] Anuse VS, Shankar K, Velmurugan R, Ha SK (2022). Compression-after-impact analysis of carbon fiber reinforced composite laminate with different ply orientation sequences. Int J Impact Eng.

[CR3] Bhudolia SK, Joshi SC (2018). Low-velocity impact response of carbon fibre composites with novel liquid Methylmethacrylate thermoplastic matrix. Compos Struct.

[CR4] Bunea M, Bria V, Silva FS, Bîrsan IG, Buciumeanu M (2021). Influence of fiber orientation and fillers on low velocity impact response of the fabric reinforced epoxy composites. Appl Compos Mater.

[CR5] Currey JD (2012). Bones.

[CR6] Dong Y, Wang F, Zhang Y, Shi X (2022). Experimental and numerical study on flow characteristic and thermal performance of macro-capsules phase change material with biomimetic oval structure. Energy.

[CR7] Dunlop JWC, Fratzl P (2010). Biological Composites. Annu Rev Mater Res.

[CR8] Ekhtiyari A, Shokrieh MM (2022). A novel rate-dependent cohesive zone model for simulation of mode I dynamic delamination in laminated composites. Compos Struct.

[CR9] Giner E, Arango C, Vercher A, Javier-Fuenmayor F (2014). Numerical modelling of the mechanical behaviour of an osteon with microcracks. J Mech Behav Biomed Mater.

[CR10] Giraudguille MM (1988). Twisted plywood architecture of collagen fibrils in human compact bone osteons. Calcif Tissue Int.

[CR11] Gustafsson A, Wallin M, Khayyeri H, lsaksson H (2019). Crack propagation in cortical bone is affected by the characteristics of the cement line: a parameter study using an XFEM interface damage model. Biomech Model Mechanobiol.

[CR12] Hamed E, Lee Y, Jasiuk I (2010). Multiscale modeling of elastic properties of cortical bone. Acta Mech.

[CR13] Hansen P, Martin RDCB (1999). 4ENF and MMB delamination characterization of S2/8552 and IM7/8552.

[CR14] Ingrole A, Trevor GA, Luca F, Donahue SW (2021). Bioinspired energy absorbing material designs using additive manufacturing. J Mech Behav Biomed Mater.

[CR15] Ismail AA, Daud R, Junoh AK, Zain NAM, Omar MI, Mansor NN (2019). Effect of lamellae thickness on the stress distribution in single osteon with the presence of lacunae. Mater Today Proc.

[CR16] Jiang H, Ren Y, Liu Z, Zhang S (2019). Microscale finite element analysis for predicting effects of air voids on mechanical properties of single fiber bundle in composites. J Mater Sci.

[CR17] Jiang H, Ren Y, Liu Z, Zhang S, Lin Z (2019). Low-velocity impact resistance behaviors of bio-inspired helicoidal composite laminates with non-linear rotation angle based layups. Compos Struct.

[CR18] Karakuzu R, Erbil E, Aktas M (2010). Impact characterization of glass/epoxy composite plates: an experimental and numerical study. Compos Part B.

[CR19] Liu D, Wagner HD, Weiner S (2000). Bending and fracture of compact circumferential and osteonal lamellar bone of the baboon tibia. J Mater Sci Mater Med.

[CR20] Liu Y, Chen B, Yin D (2017). Effects of arrangement direction and shape of osteocyte lacunae on resisting impact and micro-damage of osteon. J Mater Sci Mater Med.

[CR21] Liu Y, Li A, Chen B (2019). Effects of structure characteristics of osteocyte lacunae on squeeze damage resistance of osteons. Cells Tissues Organs.

[CR22] Luo Y, Ren Y, Shu Y, Mao C, Zhou Z, Bi ZM (2022). Cutting behavior of cortical bone in different bone osteon cutting angles and depths of cut. Chin J Mech Eng.

[CR23] Pingulkar H, Mache A, Munde Y, Siva I (2021). A comprehensive review on drop weight impact characteristics of bastnatural fiber reinforced polymer composites. Materials Today Proc.

[CR24] Rahimizadeh A, Sarvestani HY, Li L, Robles JB, Backman D, Lessard L, Ashraf B (2021). Engineering toughening mechanisms in architectured ceramic-based bioinspired materials. Mater Des.

[CR25] Reznikov N, Shahar R, Weiner S (2014). Bone hierarchical structure in three dimensions. Acta Biomater.

[CR26] Sayam A, Rahman ANMM, Rahman MS, Smriti SA, Ahmed F, Rabbi MF, Hossain M, Faruque MO (2022). A review on carbon fiber-reinforced hierarchical composites: mechanical performance, manufacturing process, structural applications and allied challenges. Carbon Letters.

[CR27] Sharma V, Borkute G, Gumfekar SP (2022). Biomimetic nanofiltration membranes: Critical review of materials, structures, and applications to water purification. Chem Eng J.

[CR28] Veerakumar VGS, Shanmugavel BP, Paskaramoorthy R, Harish S (2021). The influence of graphene nanoplatelets on the tensile and impact behavior of glass-fiber-reinforced polymer composites. J Mater Eng Perform.

[CR29] Vercher A, Giner E, Arango C, Tarancon JE, Fuenmayor FJ (2014). Homogenized stiffness matrices for mineralized collagen fibrils and lamellar bone using unit cell finite element models. Biomech Model Mechanobiol.

[CR30] Vercher-Martínez A, Giner E, Arango C, Fuenmayor FJ (2014). Influence of the mineral staggering on the elastic properties of the mineralized collagen fibril in lamellar bone. J Mech Behav Biomed Mater.

[CR31] Wang Z, Sun Y, Wu H, Zhang C (2018). Low velocity impact resistance of bio-inspired building ceramic composites with nacre-like structure. Constr Build Mater.

[CR32] Wang Y, Naleway SE, Wang B (2020). Biological and bioinspired materials: Structure leading to functional and mechanical performance. Bioactive Mater.

[CR33] Wang H, Wang C, Hazell PJ, Wright A (2021). Insights into the high-velocity impact behaviour of bio-inspired composite laminates with helicoidal layups. Polym Testing.

[CR34] Wang C, Su D, Xie Z, Wang H, Hazell PJ, Zhang Z, Zhou M (2022). Dynamic behaviour of bio-inspired heterocyclic aramid fibre-reinforced laminates subjected to low-velocity drop-weight impact. Compos A Appl Sci Manuf.

[CR35] Wei Z, Xu X (2020). FEM simulation on impact resistance of surface gradient and periodic layered bionic composites. Compos Struct.

[CR36] Yadav RN, Uniyal P, Sihota P, Kumar S, Dhiman V, Goni VG, Sahni D, Bhadada SK, Kumar N (2021). Effect of ageing on microstructure and fracture behavior of cortical bone as determined by experiment and extended finite element method (XFEM). Med Eng Phys.

[CR37] Yin D, Chen B, Lin S (2021). Finite element analysis on multi-toughening mechanism of microstructure of osteon. J Mech Behav Biomed Mater.

[CR38] Zhang Y, Chen P (2021). An improved methodology of constructing inter-fiber failure criteria for unidirectional fiber-reinforced composites. Compos A Appl Sci Manuf.

[CR39] Zhang X, Zhou R, Chen J, Lu Y, Cao D (2020). Influence of Low-velocity Impact on Damage Behavior of Carbon Fiber-reinforced Composites. J Wuhan Univ Technol Mater Sci Ed.

